# Burden of iron overload among non-chronically blood transfused preschool children with sickle cell anaemia

**DOI:** 10.4314/ahs.v21i2.34

**Published:** 2021-06

**Authors:** Akodu Samuel Olufemi, Adekanmbi Abiodun Folashade, Ogunlesi Tinuade Adetutu

**Affiliations:** Department of Paediatrics, Olabisi Onabanjo University Teaching Hospital, Sagamu, Ogun, Nigeria

**Keywords:** Sickle cell anemia, iron overload, serum ferritin, transferrin saturation, elevated iron

## Abstract

**Background:**

Sickle cell disease is the commonest genetic disorder of haemoglobin due to inheritance of mutant haemoglobin genes from both parents. The disorder is characterized by chronic haemolysis which results in increased availability of iron from red blood cell destructions.

**Objective:**

To determine the prevalence of iron overload among non-chronically blood transfused preschool children with sickle cell anaemia.

**Methods:**

Serum ferritin was assayed and transferrin saturation derived in 97 steady state sickle cell anaemia children. Elevated iron stores were defined as serum ferritin level >300ng/ml, and transferrin saturation >45%.

**Results:**

Serum ferritin level was greater than 300 mg/ml in 14 (14.4%) subjects and transferrin saturation >45% in six (6.2%) subjects with sickle cell anaemia. The prevalence of iron overload was 20.6%. The prevalence of iron overload was higher among subjects in older age group, female, with history of blood transfusion, and with single blood transfusion session

**Conclusion:**

Iron overload is prevalent in older children; the number of blood transfusion sessions notwithstanding. Regular assessment of serum ferritin is recommended.

## Introduction

Sickle cell anaemia is the most common inherited disorder of hemoglobin characterized by chronic haemolytic anaemia as a result of inheritance of mutant hemoglobin genes from both parents.^1^ In Nigeria, the prevalence of the sickle cell trait is about 25% of the population.^2^ Sickle cell anaemia patients often require multiple red blood cell (RBC) transfusions. The frequency of blood transfusion among children with sickle cell anaemia is now less as a result of improved management in recent years.^3^ In addition, frequency of blood transfusion and need for transfusion varies among children with sickle cell anemia.

In humans, total iron body stores is maintained within normal limit by adequate adjustment of intestinal iron absorption, since no excretory mechanisms exist.^4^ The mechanisms by which iron overload develops in sickle cell anaemia include: chronic haemolysis which results in increased availability of iron directly from lysed red cells, increased absorption of iron from the gastrointestinal tract and the high load of iron provided by multiple blood transfusions.^5^ If iron is allowed to accumulate it causes tissue damage, with hepatic, endocrine and cardiac failure, the latter resulting in death if untreated. Heart disease is the commonest cause of death due to iron overload.^6^

There has been more research into iron deficiency in patients with sickle cell anaemia but the iron overload burden in patients with sickle cell anaemia is yet to be extensively investigated locally. Iron chelators are not usually offered to sickle cell anaemia patients due to cost. Improved management in recent times has made blood transfusion less frequent. The diagnosis of iron overload requires sequential steps such as clinical evaluation, biochemical testing and assessment of total body iron to reach the correct diagnosis but unfortunately due to cost this is not routinely done in the care for children with sickle cell anaemia in our setting.

In general there is fear of iron overload resulting from an added effect of multiple blood transfusions but with improving management in recent years blood transfusions are less frequent and the threat of iron overload may not be real. In addition, frequency and need for blood transfusion are not uniform for all children with sickle cell anaemia. Evaluation for iron overload in children with sickle cell anaemia is important, as it could contribute to the growth impairment seen in them and lead to death. The objective of the study was to evaluate the burden of iron overload among pre-school children with sickle cell anaemia who were in steady state. It is therefore expected that the information derived from the study would help the clinicians to provide timely medical management for children with sickle cell anaemia. The study would thus add to local data.

## Subjects and Methods

The study was a prospective, cross-sectional one conducted at a pediatric sickle cell clinic of a tertiary health institution and one of the main referral facilities providing both general and specialist pediatric care for inhabitants of one of the largest cities in West Africa. The study duration was three months from December 2009 to February 2010. Subjects who met the study criteria were recruited: (1) subjects screened for presence of HbSS by haemoglobin electrophoresis (2) steady state defined as absence of any crisis in the preceding four weeks and absence of any symptoms or signs attributable to an acute illness7 (3) age six months to five years. None of the recruited subjects had been transfused or has been taking iron supplementation during a three month interval before study.

The minimum sample size was determined using the Fisher's formula^8^ stated below:

. n = z2pq/d^2^

Where:

n = minimum sample size

z = percentage point of standard normal distribution curve which corresponds to 95% confidence interval. It is equal to 1.96 (at 95% confidence limits) in a twotailed test.

p = prevalence rate of iron overload for sickle cell anaemia in Nigeria by Odunlade et al9 is 33.3%.

q = complimentary probability; q = 1-0.333 = 0.667.

d = degree of precision at 90% confidence limit; d = 10% = 0.1

therefore, the minimum required sample size was 85.

Approximately 7 mL of venous blood was collected from each of the subjects, 2ml and 5ml were transferred in a Na-EDTA containing and plain vacuum tubes for haemoglobin and serum studies respectively. The labeled tubes were transported to the Research Laboratory Paediatrics Department, LASUTH, placed in a cool box containing ice-packs within 8 - 12 hours of collection. Sheets of black plastic were used to protect the samples from light at all times. The fresh blood samples collected in Na-EDTA containing tubes were used for haemoglobin concentration estimation. Following centrifugation of the sample in the plain vacuum tube, the serum was separated and stored at minus 20ºC until ready for iron profile assay. Human ferritin enzyme immunoassay test kit (Diagnostic Automation, USA) was used to measure the serum ferritin. Additional iron profile parameters measured from the serum included serum iron concentration, and unsaturated iron binding capacity. Serum levels of these biochemical parameters were determined according to standard laboratory procedures. The transferrin saturation and total iron binding capacity were determined using these measured parameters.

Ferritin levels and transferrin saturation are simple tests that have been used for many decades to diagnose and monitor iron overload.^10^ Iron overload was defined as serum ferittin value greater than 300ng/ml9 or transferrin saturation greater than 45%^11^, ^12^. Scheme proposed by Oyedeji.^13^ was used to classify subjects into one of five classes (I – V) in descending order of social privilege using occupation and educational attainment of parents. Classes I and II were grouped together as upper social stratum, class III was taken as the middle stratum and classes IV and V as lower social strata.

The study design was approved by the ethics and research committee of LASUTH prior to the commencement of research. Subjects were recruited following detailed explanation about the research. All recruitments were strictly voluntary and backed by written informed consent.

Statistical Package for Social Sciences (SPSS for Windows Version 17.0) was used for data analysis. The data were presented as percentage and mean ± standard deviation (±SD). Student t-test was used to compare the mean values. The relationship between continuous and categorical variables was assessed using Pearson correlation coefficient (r) and Chi-square (χ2) as applicable. Logistic regression analysis was performed to calculate Odds ratio (OR) and 95% confidence intervals for all the predictor variables with p value < 0.05 in the Chi square analysis to assess their strength of association with the prevalence of iron overload. Statistical significance was set at p < 0.05.

## Results

### Mean values of iron overload indicators among study subject

A total of 97 children with sickle cell anaemia in steady state were recruited with male to female ratio of 1.02:1.00. Overall, the age of the subjects ranged from seven months to five years, with a mean of 30.61 (±15.97) months and a median of 25.00 months.

In table 1, the mean values of iron status indicators among study subjects are shown. The mean serum iron value was comparable among both age categories. The mean TIBC was higher among ≤2 years age category compared to their >2 – 5 years counterpart but this difference was not significant (p = 0.262). The mean serum ferritin and transferrin saturation values were higher among subjects >2 – 5 years than subjects ≤2 years but the observed difference was not significant (p = 0.256, 0.241 respectively).

**Table I:** Iron overload indicators among study subject

Iron Parameters	≤2 years	>2 – 5 years	p-value
Serum Iron (mcg/dL)			0.957
Mean (SD)	67.68 (46.63)	67.15 (37.41)	
Median	55.00	64.00	
Range	10.00 – 186.00	8.00 – 164.00	
TIBC (mcg/dL)			0.262
Mean (SD)	273.85 (95.15)	250.54 (77.10)	
Median	292.00	254.50	
Range	46.00 – 452.00	47.00 – 418.00	
Serum ferritin (ng/dL)			0.256
Mean (SD)	172.48	200.62	
Median	(100.16)	(106.37)	
Range	155.00	210.00	
	31.00 – 350.00	10.00 – 360.00	
Transferrin saturation (%)			0.241
Mean (SD)	23.69 (12.40)	27.37 (12.82)	
Median	20.40	28.24	
Range	3.45 – 48.69	1.91 – 49.34	

### Distribution of individual criteria for diagnosing iron overload among study subjects

In table 2, the prevalence of iron overload based on individual criteria is shown. A criterion of serum ferritin greater than 300ng/ml was found in 14.4% of sickle cell anaemia subjects. The adopted definition of iron overload included a second criterion – transferrin saturation greater than forty-five percent. Using transferrin saturation greater than forty-five percent criterion, six subjects was identified as having iron overload and they were different from the subjects using the serum ferritin greater than 300ng/ml criterion.

**Table II:** Distribution of individual criteria for diagnosing iron overload among study subjects

Iron Overload Criteria	Number Affected	%
Serum ferritin > 300ng/dL	14	14.4
Transferrin saturation >45%	6	6.2
Serum ferritin > 300ng/dL or Transferrin saturation >45%	20	20.6

### Distribution of iron overload among study subjects according to demographic and selected clinical characteristics

The distributions of study subjects with iron overload according to demographic and selected clinical characteristics are shown in Table 3. The overall prevalence of iron overload was 20.6%. In the older age group the prevalence of iron overload was higher compared with the younger age group. More females than males have iron overload. The prevalence of iron overload was higher among subjects with history of blood transfusion compared to subjects without history of blood transfusion. Subjects with single blood transfusion session had a higher prevalence rate of iron overload than subjects who had multiple blood transfusion session while the prevalence rate was comparable among subjects who belonged to the lower socioeconomic strata with those from other socioeconomic strata. However, all these differences were not significant (p > 0.05).

**Table III:** Distribution of iron overload among study subjects according to demographic and selected biological characteristics

		Iron Overload n (%)	p-value
Age group (years)			0.453
	≤2	8 (16.7)	
	>2 – 5	12 (24.5)	
Gender			0.139
	Male	7 (14.3)	
	Female	13 (27.1)	
History of			0.599
blood transfusionr	Yes	8 (24.2)	
	No	12 (18.8)	
			0.875
Number of blood	0	0 (0.0)	
transfusion			
	1	7 (30.4)	
			0.949
	>1	1 (10.0)	
Socioeconomic strata			
	Lower	3 (20.0)	
	Others	17 (20.7)	

### Comparisons of mean values of iron overload indicators between subjects with and without iron overload

In table 4, the mean values of iron overload indicators between study subjects with and without iron overload are shown. The mean serum iron value was significantly higher among subjects with iron overload compared with those without iron overload. Similarly, the mean TIBC value was significantly higher among subjects with iron overload compared with those without iron overload. The mean serum ferritin and transferrin saturation values were higher among subjects with iron overload than subjects without iron overload but the observed difference was not significant (p = 0.109, 0.236 respectively).

**Table IV:** Comparisons of mean values of iron overload indicators between subjects with and without iron overload

Iron Parameters	Iron Overload Mean (SD)	No Iron Overload Mean (SD)	p-value
Serum Iron (mcg/dL)	84.00 (52.60)	62.83 (36.18)	0.028
TIBC (mcg/dL)	285.31 (119.61)	251.43 (70.58)	0.002
Serum ferritin (ng/dL)	294.75 (86.00)	161.92 (90.36)	0.109
Transferrin saturation (%)	31.50 (14.33)	24.43 (11.87)	0.236

## Discussion

Iron overload has been reported to be common in subjects with recurrent transfusion, and the degree of iron overload increase with the rate of transfusions.^14^, ^15^ Our findings are significant for several reasons. First, we do not believe that magnitude of iron overload in subjects with sickle cell anaemia with less frequent blood transfusions has been previously well described in Nigeria. Secondly, given the prevalence of iron overload-induced medical problems in patients with sickle cell anaemia, as well as the improvement in the management of subjects with sickle cell anaemia in recent years making blood transfusions to be less frequent, there are likely to be undiagnosed cases of iron overload in this population since routine serum iron estimation is not practice in our setting.

The overall prevalence of iron overload among children with sickle cell anaemia in the present study was 20.6%. The reported prevalence value from the current study was lower than the 28.6% reported by Ray et al16 and33.3% by Odunlade et al^9^ among children with sickle cell disorder in West Bengal, India aged 3 – 18 years and 1 – 15 years in Southwestern Nigeria. The observed difference is probably an effect of diagnostic criteria. The Indian study employed the use of serum values of iron and ferritin greater than 184µg/dL and 140mg/dL respectively while the Southwestern Nigeria study employed the use of serum ferritin greater than 300mg/dL alone. In addition, the observed difference may be an effect of sample size Small sample size is known to produce exaggerated high mean values. These observed differences may also be due to disparity in rate of chronic haemolysis, and genetic variation in gastrointestinal iron absorption among the study subjects which is incomparable.

The current study revealed a higher prevalence of iron overload among subjects older than two years of age than in the younger age group. This may be due to the fact that the older subjects would be more affected because they have had chronic haemolysis for a longer period. This observed difference in prevalence of iron overload based on age group did not reach significant level. It is possible that the lower numbers of affected children with iron overload was responsible for the lack of significance.

In variance to reported female preponderance from this present study a male preponderance was reported by Makulo et al^17^ among Congolese children with sickle cell anaemia aged 2 – 18 years. There is no information available regarding the influence of gender on the burden of iron overload among children with sickle cell anaemia.

A comment on the place of blood transfusion in relation to iron overload among study subjects is desirable at this point. It was observed that iron overload was found more in children with sickle cell anaemia who received blood transfusion. Previous study has attributed iron overload in children with sickle cell anaemia to increased red blood cell turnover and multiple blood transfusions.^10^ The subjects with one previous blood transfusion was observed to have higher rate of iron overload compared with counterparts who had received two or more blood transfusions in the past. However, this observed difference was not statistically significant. This report contrast with earlier observations in which children with multiple blood transfusions are associated with higher burden of iron overload.^10^ The reason for this is not quite clear; however it might be attributed to individual disparity in rate of haemolysis and genetic variation in gastro-intestinal iron absorption among the study subjects which is incomparable. This may also be direct effect of heterozygous hemochromatosis among the study subjects. The fact that the current study made no attempt to detect/exclude heterozygous hemochromatosis among the study subjects is in fact an important limitation of this study.

The extent to which serum ferritin criteria identified subjects with iron overload varied compare with the transferrin saturation criteria. The use of serum ferritin identified almost twice the numbers by the transferrin saturation with the identified subjects in each category completely different. This may be due to the fact that elevations in transferrin saturation typically precede a rise in serum ferritin in patients with iron overload as reported by Lam.^18^

The present study revealed that both the mean serum iron and TIBC were significantly higher in subjects with iron overload than those without iron overload. Also, a higher but not significantly different mean serum transferrin and transferrin saturation in subjects with iron overload than those without iron overload. The trend of higher serum iron, TIBC, serum ferritin and transferrin saturation in children with sickle cell anaemia having iron overload is consistent with screening findings in subjects with hemochromatosis.^19^, ^20^. It will have been necessary to exclude heterozygous hemochromatosis in the study subjects; however, the diagnosis of hemochromatosis in children within our setting is difficult or impossible because of lack of resources for genetic testing and to the best of our knowledge hemochromatosis has never been reported in our setting.

Overall, non-chronically blood transfused preschool children with sickle cell anaemia also suffer from iron overload like those with multiple blood transfusions. The study revealed the need for periodic determination of iron over load in children with sickle cell anaemia in steady state that are not chronically transfused using either serum ferritin or transferrin saturation assay. It would have been desirable to perform the estimation of the acute phase reactants levels such as C-reactive protein (CRP) since the observed elevated serum ferritin may also be a false positive due to inflammation instead of iron overload. Another limitation is the failure to perform liver biopsy which is an invasive technique to assess iron overload or imaging techniques such as magnetic resonance and Superconducting Quantum Interference Device (SQUID) which have been reported to provde a good correlation between iron tissue and potential organic damage.
